# Transcriptional profiles of valvular interstitial cells cultured on tissue culture polystyrene, on 2D hydrogels, or within 3D hydrogels

**DOI:** 10.1016/j.dib.2015.11.017

**Published:** 2015-11-17

**Authors:** Kelly M. Mabry, Samuel Z. Payne, Kristi S. Anseth

**Affiliations:** a Department of Chemical and Biological Engineering, University of Colorado at Boulder, Boulder, CO 80309, USA; b Howard Hughes Medical Institute and the BioFrontiers Institute, University of Colorado at Boulder, Boulder, CO 80309, USA

## Abstract

Valvular interstitial cells (VICs) actively maintain and repair heart valve tissue; however, persistent activation of VICs to a myofibroblast phenotype can lead to aortic stenosis (Chen and Simmons, 2011) [1]. To better understand and quantify how microenvironmental cues influence VIC phenotype, we compared expression profiles of VICs cultured on/in poly(ethylene glycol) (PEG) gels to those cultured on tissue culture polystyrene (TCPS), as well as fresh isolates. Here, we present both the raw and processed microarray data from these culture conditions. Interpretation of this data can be found in a research article entitled “Microarray analyses to quantify advantages of 2D and 3D hydrogel culture systems in maintaining the native valvular interstitial cell phenotype” (Mabry et al., 2015) [2].

**Specifications Table**

**Table:** 

Subject area	*Biology*
More specific subject area	*Biomaterials*
Type of data	1. *Raw microarray data* 2. *Processed and annotated microarray data in spreadsheet*
How data was acquired	*Affymetrix PorcineGene 1.0ST microarray chips*
Data format	*Raw data in .CEL files and processed data in Excel spreadsheet*
Experimental factors	*Comparison of freshly isolated valvular interstitial cells (VICs) to VICs cultured on tissue culture polystyrene, 2D hydrogel substrates, or encapsulated within 3D hydrogel matrices.*
Experimental features	*Peptide-functionalized poly(ethylene glycol) hydrogels permit controlled presentation of mechanical and biochemical matrix cues to VICs and prevent the activation of VICs to the activated myofibroblast phenotype associated with valve disease. The transcriptional profile of VICs cultured in 2D and 3D hydrogel systems is compared to traditional culture on tissue culture polystyrene and to freshly isolated VICs.*
Data source location	*Samples collected in Boulder, CO, USA. Microarrays performed in Aurora, CO, USA.*
Data accessibility	*Data is available with this article.*

## Value of the data

• With access to this data, other researchers can further investigate how the dimensionality of the cell culture environment influences VIC phenotype and determine whether similar effects are observed in other cell types.
• Comparisons between the transcriptional profiles of VICs and other fibroblast populations could lead to insight into the fibroblast-to-myofibroblast transition and to the progression of and possible treatment of fibrotic diseases.
• For the future investigation of pathways of interest, researchers could determine if the relevant genes are expressed at similar levels when cells are cultured in one of these three culture platforms (TCPS, 2D hydrogel, 3D hydrogel) compared to the freshly isolated VICs.

## 1 Data

The raw data files (.CEL) that were used in the analysis are available in Supplementary information, and the file names are identified in Table 1. Three biological replicates were performed using VICs from separate pools of porcine hearts. The processed data, which gives the expression levels for each probe set, is also included in Supplementary information (Supplementary Table 1).

## 2 Experimental design, materials and methods

VICs were isolated from aortic valve leaflets of fresh porcine hearts (Hormel) using a previously described protocol [2], [3] A fraction of these freshly isolated cells were immediately flash-frozen in TriReagent (Sigma-Aldrich) to serve as the freshly isolated VIC control. The remaining cells were re-suspended in growth media composed of Media 199 (Life Technologies) supplemented with 15% fetal bovine serum (FBS, Life Technologies), 1% penicillin–streptomycin and 0.5 μg/mL fungizone. These VICs were grown to ~80% confluency on TCPS prior to use in experimental conditions.

Hydrogels were formed via a photoinitiated thiol-ene reaction that has been described previously [4], [5] 5 wt% 8-arm PEG-norbornene was crosslinked with a dithiol-containing, matrix metalloprotease (MMP)-degradable peptide KCGPQG↓IWGQCK (American Peptide Company, Inc.) in the presence of 2 mM CRGDS adhesive peptide (American Peptide Company, Inc.) at a ratio of 0.55 thiols per norbornene. The photoinitiator lithium phenyl-2,4,6-trimethylbenzoylphosphinate (LAP) was employed at a concentration of 1.7 mM. Polymerization was performed in phosphate buffered saline (PBS, Life Technologies). VICs secrete the MMPs to which the crosslinking peptide sequence is susceptible [1], which permits local degradation of the matrix. Non-stoichiometric ratios of the thiol and –ene functionalities were used to control the final crosslinking density, and ultimately, the gel connectivity and shear modulus to permit cell spreading in cell-laden hydrogels within 48 h.

2D hydrogels were fabricated on glass coverslips that had been thiolated by vapor deposition of 3-(mercaptopropyl) trimethoxysilane in an 80 °C oven to enable covalent attachment of the gels to the coverslips. First, the monomer solution was pipetted onto a SigmaCote (Sigma-Aldrich) treated glass slide and covered with a thiolated coverslip such that the final gel thickness was ~100 μm. VICs were then seeded on 2D hydrogels at 25,000 cells/cm^2^. For 3D hydrogels, 10 million cells/mL were suspended in the monomer solution, and 29 μL of the cell-monomer solution were added to a mold (5 mm diameter) placed on a SigmaCote treated glass slide. All hydrogels were polymerized by exposure to UV light (~2 mW/cm^2^ at 365 nm) for 3 min VICs were seeded on TCPS at a cell density of 12,500 cells/cm^2^. Experiments were performed in low-serum (1% FBS) media supplemented with 1% penicillin–streptomycin (Life Technologies) and 0.5 μg/mL fungizone (Life Technologies) in a 37 °C incubator with 5% CO_2_. The Young׳s modulus of the swollen hydrogels was determined to be 390 Pa by performing frequency and strain sweeps using a DHR3 rheometer (TA Instruments) with a parallel plate geometer.

RNA was isolated from VICs 48 h after seeding or encapsulating using TriReagent (Sigma-Aldrich) according to the manufacturer׳s instructions. Two sequential extractions with 1-bromo-3-chloropropane (Sigma-Aldrich) were followed by precipitation with 2-propanol (Sigma-Aldrich). RNA pellets were washed twice with 75% ethanol (Sigma-Aldrich) and re-suspended in water. A Nanodrop ND-1000 Spectrophotometer was used to assess RNA concentration and quality. Only samples with a concentration greater than 100 ng/μL, 260/280≥1.8, and 260/230≥1.8 were used in the microarray experiments. RNA quality assessment and microarrays were performed by the Genomics and Microarray Core and University of Colorado at Denver. RNA integrity numbers were determined using a TapeStation 2200 (Agilent) and a cutoff RIN of 7 was implemented. Samples were hybridized to Affymetrix Porcine Gene 1.0 ST arrays. Data was processed using Expression Console (Affymetrix) for gene-level normalization, probe set summarization and quality control. Then, Transcriptome Analysis Console (Affymetrix) was used to evaluate differential gene expression.

## Figures and Tables

**Table 1:** Naming of .CEL files.

Sample ID	Condition
2DB	Seeded on 2D hydrogel substrate
2DC
2DD
3DB	Encapsulated within 3D hydrogel
3DC
3DD
TCPSB	Seeded on TCPS
TCPSC
TCPSD
FB	Freshly isolated
FC
FD

## Footnotes

Appendix A Supplementary data associated with this article can be found in the online version at doi:10.1016/j.dib.2015.11.017.
